# Supplementary material for: Diode-Like Current Leakage and Ferroelectric Switching in Silicon SIS Structures with Hafnia-Alumina Nanolaminates
Source: Nanomaterials (Basel). 2021 Jan 22;11(2):291. doi: 10.3390/nano11020291 (PMC7912112; doi:10.3390/nano11020291)
Supplement: Supplementary file 1 [file nanomaterials-11-00291-s001.pdf]

Supplementary Materials

Leakage currents and ferroelectric switching in silicon SIS structures with high-k HfO_2 and Al_2O_3 nanolaminates

*Vladimir Popov *, Fedor Tikhonenko , Valentin Antonov , Ida Tyschenko , Andrey Miakonkikh , Sergey Simakin, Andrey Lomov, Konstantin Rudenko*

Thin nanolaminated aluminum oxide (Al_2O_3)/hafnium oxide (HfO_2) films, as well as single Al_2O_3 and HfO_2 layers, were grown as insulators for SIS structures by the plasma-enhanced atomic layer deposition (PEALD) technique on a silicon substrate before the wafer bonding and Si layer transfer on it (**Figure S1**), according to the invention [S1]. The pair of wet cleaned wafers were placed in the vacuum chamber and heated in vacuum up to 200°C for SFS or at 400°C for SOF structures in order to remove the physically absorbed molecules from the wafer surfaces before bonding and to suppress the large stress generation during the followed thermal splitting of hydrogen implanted Si wafers. Using the implanted hydrogen transfer of only Si layers allows avoiding the defect generation in the high-k stack at the H implantation and subsequent thermal treatments.

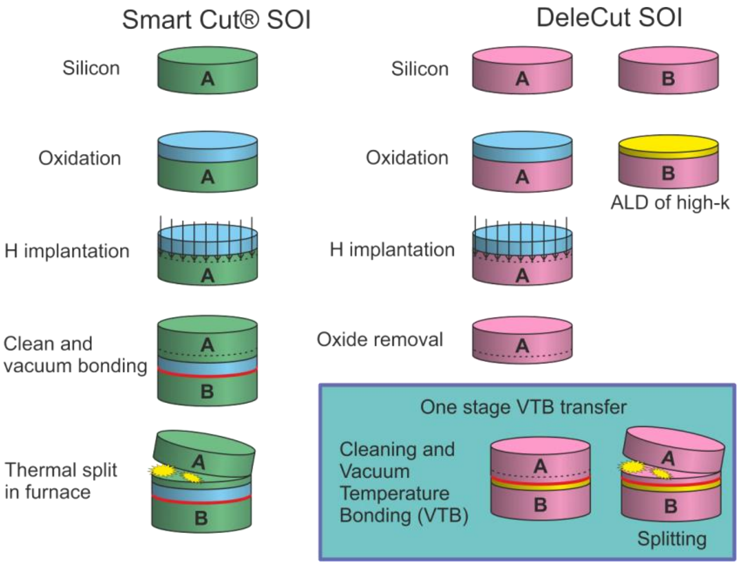

Figure S1. Two SIS structure fabrication processes used in the investigation.

The asymmetric HfO_2/Al_2O_3 stack and *vice versa* clearly show the similar n-p or p-n behavior (Figure S2). The direct-to-reverse branch current ratios of I-V leakage curves does not strongly depend on the total layer thickness and decrease of bonding interface positions (Figure S3) or increase (not shown here) of the rectification ratio, respectively.
The space xharge region (SCR) origin is due to larger negative charges of dipoles at the SiO_2/Al_2O_3 interface in the top interlayer silica than to negative charges of dipoles at the HfO_2/SiO_2 interface in the bottom interlayer silica [S2]. The difference in the dipole densities on both sides of the dielectric forms a band bending in silicon electrodes similar to the p-n junction.

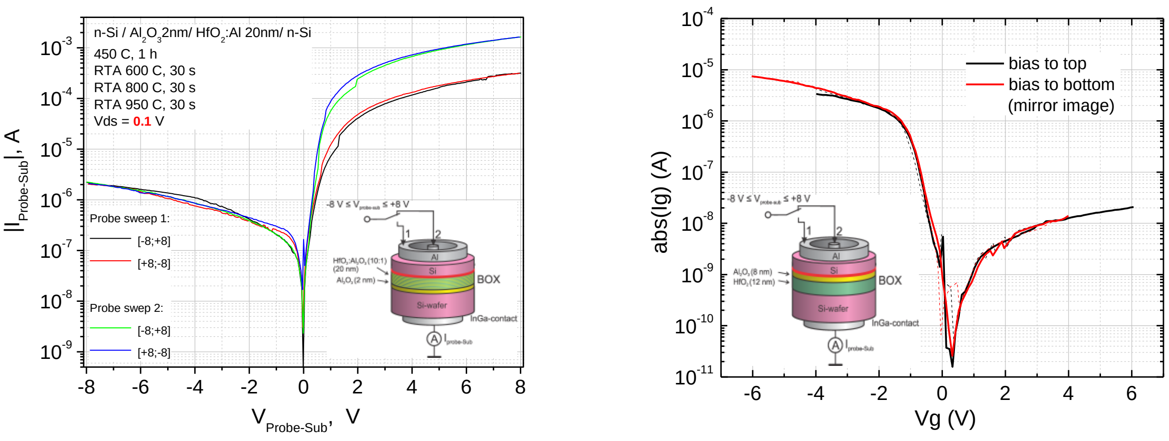

(**a**)

(**b**)

Figure S2. (**a**) Diode-like I-V curves at the source current – gate voltage (Probe sweep 2) and drain current – gate voltage (Probe sweep 1) contacts for n-SIS pseudo-MOSFETs on the structures after the RTA at 950 ℃ with different high-k BOX layers: (a) 500 nm n-Si/HfO_2:Al 20nm/Al_2O_3 2nm/n-Si-substrate; (**b**) 500 nm n-Si/Al_2O_3 8nm/HfO_2 12nm/ n-Si-substrate. The bias voltages are in the range Vg = - 8 + 8 V.

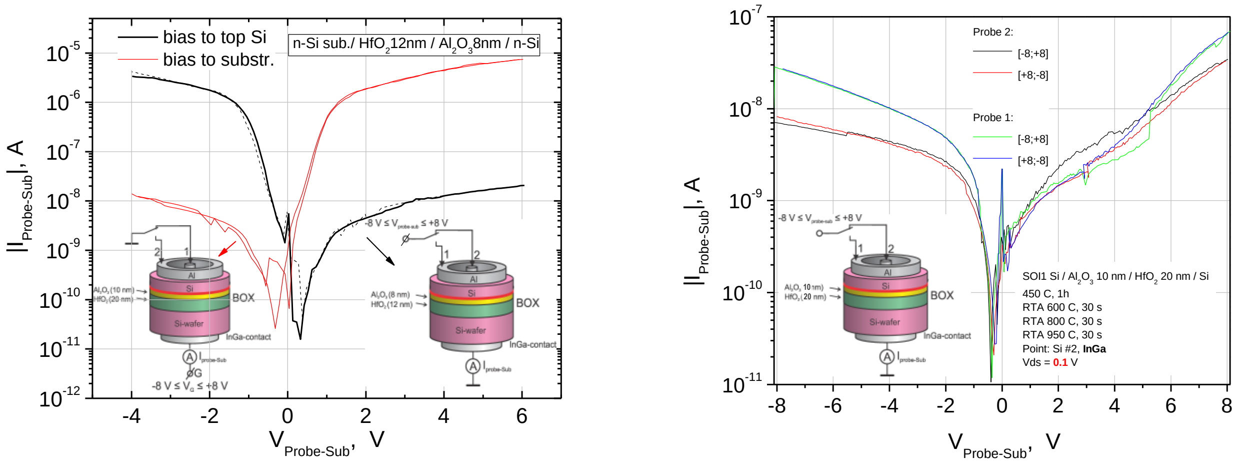

(**a**)

(**b**)

Figure S3. (**a**) Diode-like I-V curves at the source current – gate voltage (Probe sweep 2) and drain current – gate voltage (Probe sweep 1) contacts with the bias voltage in the range Vg = -8 + 8 V on the drain or source (Probe 1 or 2) for n-SIS Corbino structures after the RTA at 950 ℃ with different high-k BOX layers: 500 nm n-Si/Al_2O_3 8nm/HfO_2 12nm/ n-Si-substrate **(a)**, 500 nm n-Si/Al_2O_3 10nm/HfO_2 20nm /n-Si-substrate (**b**).

In **Figure S4** are the distribution profiles of the main atoms in the n-SIS structure after the furnace annealing at 800 ^oC for 1 hour. It includes the profiles in the Si (500 nm) film, the buried oxide (BOX) layer of high-k stack HfO_2(6 nm)/Al_2O_3(8 nm) /HfO_2(6 nm) and part of the substrate obtained by the secondary ion mass spectrometry (SIMS) of negatively charged ions with the TOF.SIMS 5 (IONTOF) facility. Sputtering was carried out with primary Cs^+ ions with the energy of 1 keV to the depth of 570 nm (Fig.S2a) and to the depth of 65 nm (Fig.S2b). The depth resolution was better than 4 nm.

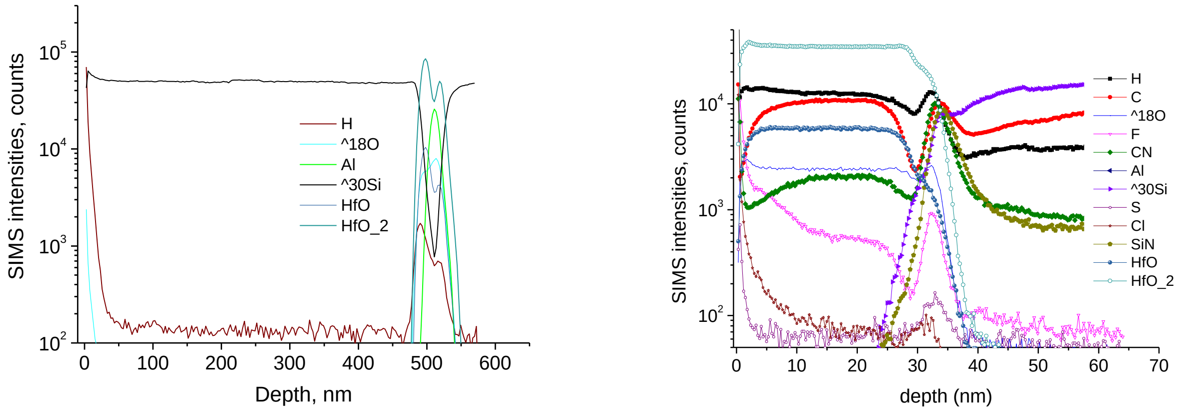

Figure S4. SIMS atom distribution profile signals in the n-SIS structure, including the top Si film (500 nm), BOX layer of high-k stack $HfO_2/Al_2O_3/HfO_2$ (20 nm) and the Si substrate after the furnace annealing at 800°C for 1 hour (a); the same but with the 30 nm HfO_2 layer after the furnace annealing at 1000°C for 1 hour and removing the upper Si layer in boiling ammonia (b).

A small hysteresis also was observed for high-k stack $HfO_2/Al_2O_3/HfO_2$ (20 nm) after the annealing at 600°C for |Vg| < 1 V (**Figure S5**). The higher bias voltage demonstrates leakage current dependences as for Fouler-Nordheim on fully logarithmic scales with the slope n = 2 (**Figure S5b**).

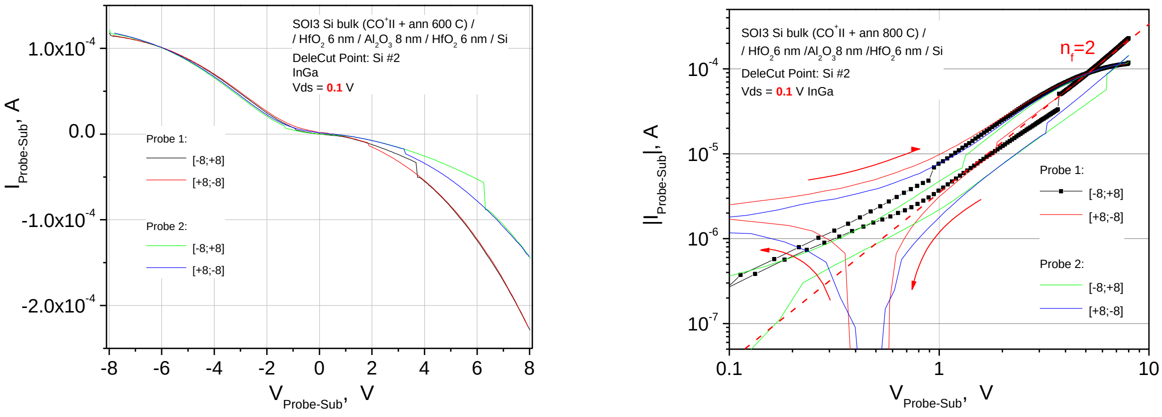

Figure S5. The I-V characteristics on the source-gate and drain-gate contacts of n-SIS pseudo-MOSFET structures in Corbino geometry with a 500 nm n-Si layer and 20 nm $HfO_2/Al_2O_3/HfO_2$ high-k stack after the annealing at 600°C 1h on the linear (a) and fully logarithmic (b) scales.

In **Figure S6** are the glancing incidence X-ray diffraction (GIXRD) spectra of the same SIS structures without the upper Si layers removed by etching in boiled ammonia dependening on the annealing temperature at 450 - 1000 ℃ during 1h. They clearly show decreasing the content of the *Pca2₁* ferroelectric phase peak at 30.5 °(black arrow) at increasing the annealing temperature.

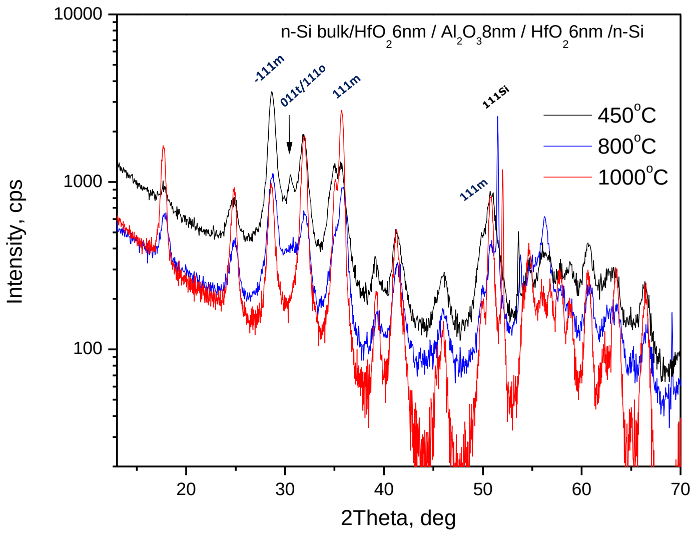

Figure S6. GIXRD spectra of the SFS sample with the 500 nm n-Si / 20 nm stack BOX of HfO_2 6 nm/ Al_2O_3 8 nm / HfO_2 6 nm/n-Si substrate (as in Fig. 5, main text) show decreasing the content of the *Pca2₁* ferroelectric phase peak at 30.5 °(black arrow) after the furnace annealing at 450 - 1000 ℃ during 1h.

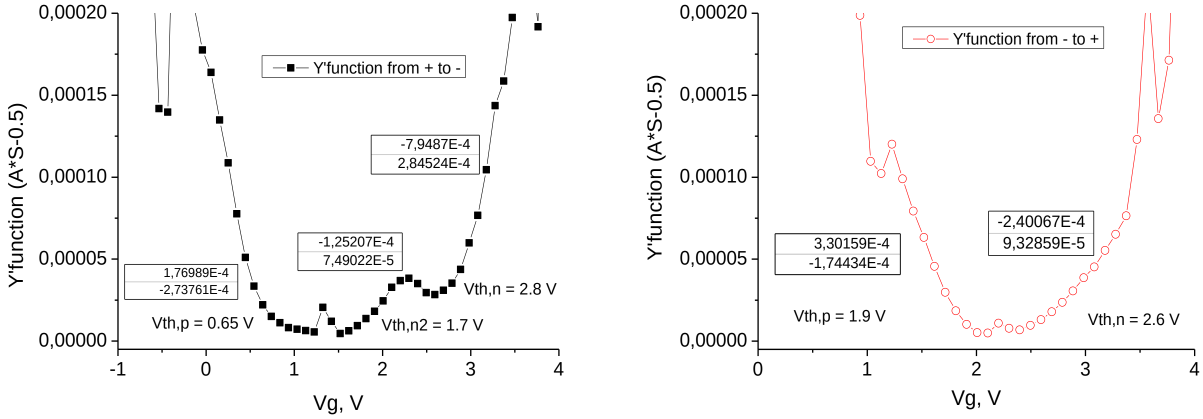

(a) **(b)**

Figure S7. The Y-function for two sweep directions or the P-up and P-down polarisation (**Figure 8, main text**). The numbers in the inserts indicate their point of intersection with the ordinate axis ($V_g = 0$ V) and the slopes of the linear extrapolation for the branches (the Y-function slope), respectively, and that gives $\Delta V_{th,p} = 1.25$ V.

The C-V and G-V curves for the n-p SFS pseudo-MOSFET mesa structures with a 500 nm n-Si layer and 20 nm $HfO_2/Al_2O_3/HfO_2$ (HAO) high-k stack transferred on the p-Si substrate are shown in Figures S8-S10 after the RTA treatment at 950°C. The strong capacity C and conductivity G switching are observed at the -3 - 0 V gate voltage interval. The capacity maximum at this interval after the P-up polarization (the high negative bias voltage is on the upper Si layer and the substrate is grounded) gives evidence for the major carrier accumulation on both sides of the insulator between n- and p-type Si layers (Figure 8b), while the further voltage increase leads to the substrate depletion and capacity decrease with the leakage increase [S3]. It is related to the P-E hysteresis and the butterfly figure for the PFM hysteresis (Figures S11a, S12b), respectively.

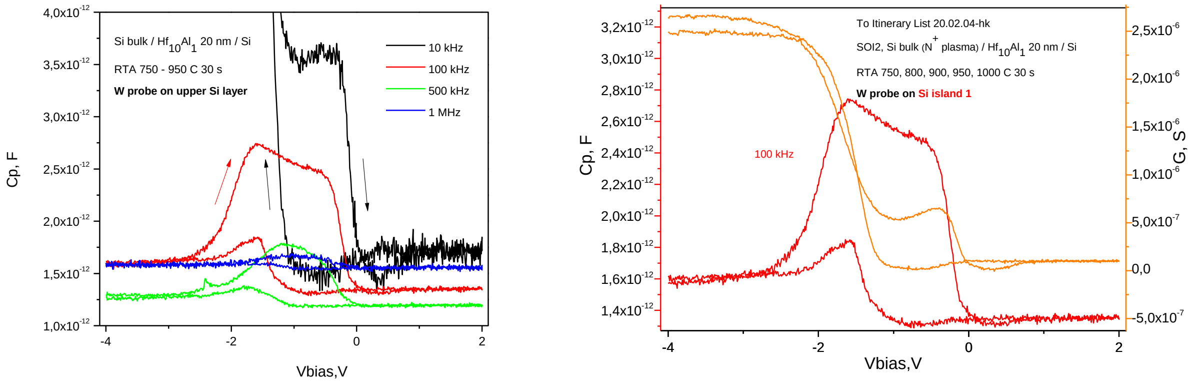

Figure S8. C-V characteristics on the source-gate contacts of the n-p HAO SFS pseudo-MOSFET mesa structures with a 500 nm layer of n-Si and 20 nm $HfO_2/Al_2O_3/HfO_2$ at different frequencies (a) and the same, but with G-V curves, for only 100 kHz (b).

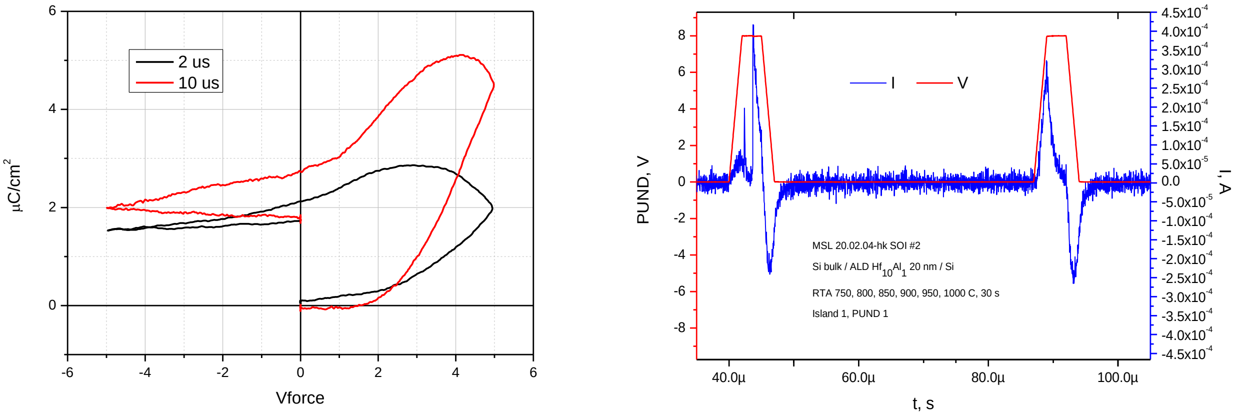

(a) (b)

Figure S9. P-V hysteresis for the n-p HAO SFS pseudo-MOSFET mesa structures with two different triangular pulse periods with the voltage amplitudes -5 +5 V (a) and PUND pulses (only the part of PUND pulse sequence with positive pulses are shown) for the source-gate characteristics after the RTA treatment at 950ºC during 30 s (b).

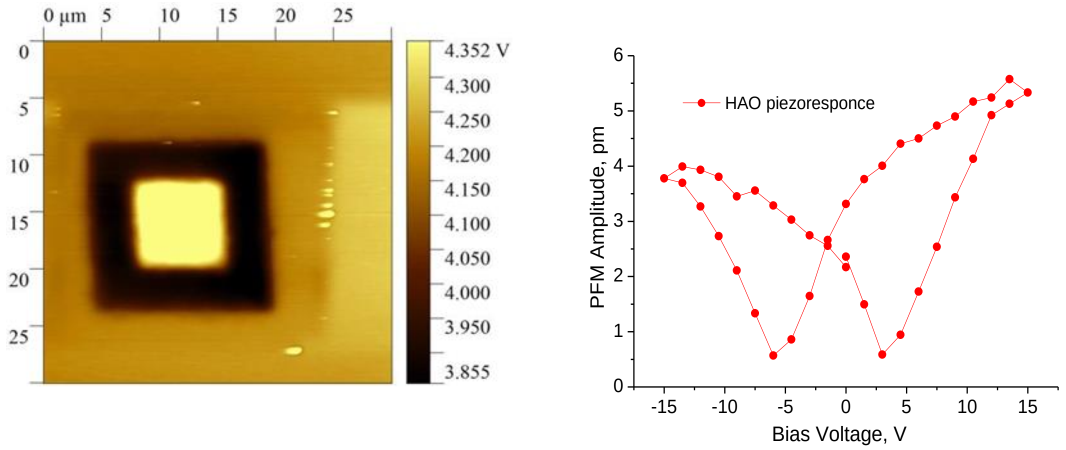

(a) (b)

Figure S10. PFM Kelvin surface potential map measured during 20 min after polarization with the voltage mask -8 V (black) and +8 V (yellow) squares (a), and the respective PFM amplitude dependence for the HAO SFS structure after after the RTA at 950ºC during 30 s and subsequent Si layer removing in boiled ammonia (b).

References

S1. Popov V.P., Tyschenko I.E. “Method of silicon-on-insulator structure creation”. *The invention PCT WO* no. 2004/064137.

S2. Fei, J., Kita, K. “Opportunity of dipole layer formation at non-SiO2 dielectric interfaces in two cases: Multi-cation systems and multi-anion systems.” *Microelectronic Engineering,* 2017, 178, p. 225-229. https://www.sciencedirect.com/science/article/pii/S0167931717302320?via%3Dihub

S3. P. J. McWhorter, P. S. Winokur, *Appl. Phys. Lett.,* **1986**, *48*, 133. https://doi.org/10.1063/1.96974
